# Long-Term Outcomes Following Cochlear Implantation: Device “Aging” and Hearing Performance

**DOI:** 10.3390/audiolres15020019

**Published:** 2025-02-24

**Authors:** Davide Soloperto, Gennaro Confuorto, Virginia Dallari, Luca Sacchetto, Marco Carner, Daniele Monzani, Riccardo Nocini

**Affiliations:** 1 Department of Otorhinolaryngology-Head and Neck Surgery, University Hospital of Modena, 41125 Modena, Italy; davide.soloperto@unimore.it; 2 Department of Otolaryngology-Head and Neck Surgery, Sassuolo Hospital, AUSL Modena, 41049 Sassuolo, Italy; confuorto.gennaro@gmail.com; 3 Department of Otolaryngology-Head and Neck Surgery, Santa Maria delle Croci Hospital, AUSL della Romagna, 48121 Ravenna, Italy; 4 Unit of Otorhinolaryngology, Head & Neck Department, University of Verona, 37134 Verona, Italy; luca.sacchetto@univr.it (L.S.); marco.carner@aovr.veneto.it (M.C.); daniele.monzani@univr.it (D.M.); riccardo.nocini@studio.univr.it (R.N.)

**Keywords:** cochlear implant, deafness, hard failure, soft failure, otology, otosurgery, revision surgery

## Abstract

Purpose: The purpose of this study was to evaluate the clinical and audiological outcomes in long-term cochlear implant (CI) users, focusing on hearing performance and device failures. Methods: A retrospective study was conducted on patients who underwent CI surgery, with a minimum follow-up of 10 years. Device survival was analyzed using Kaplan–Meier curves, and failure rates were classified as device failure explants or medical-related explants. The time to revision and causes of reimplantation were assessed. Statistical analyses included Pearson correlation for audiological outcomes, Student’s *t*-test for group comparisons, and the log-rank test for survival comparisons among implant brands. Results: In this study, data from a total of 211 CIs were collected. Fourteen major postoperative complications were reported in this series, resulting in a global major complication rate of 6.6%: 5.2% due to hard failure and 1.4% due to medical problems and soft failure. The revision rate was 4.1% for the children’s group and 10% for the adult group. The overall device survival rates were more than 96% at 10 years and 91% at 20 years. The median postoperative follow-up was 16.3 years. Moreover, a highly significant correlation was observed (r = 0.669, *p* < 0.0001) between pure tone average (PTA) thresholds at implant activation and those at the last follow-up, as analyzed using Pearson’s correlation coefficient. Conclusions: The CI is a lifelong device; however, the technology is constantly evolving. Therefore, careful patient counseling (or counseling of parents in the case of pediatric patients) is necessary. The device may wear out over time, requiring revision surgery. These data are essential for audiologists and ENT specialists when selecting patients and determining surgical indications.

## 1. Introduction

Unilateral or bilateral cochlear implant (CI) is an effective and safe surgical procedure that facilitates auditory rehabilitation in adults and children with severe to profound sensorineural hearing loss [1]. With rapid innovation in and expanding indications for CIs, the landscape of auditory rehabilitation is constantly evolving. This life-changing surgery not only enhances social interactions but also contributes to academic and professional advancements.

Despite its reliability and a very low incidence of surgical and non-surgical complications [2], revision surgery may be necessary for various reasons. As with any surgical procedure, CIs can result in post-operative complications, ranging from abnormal auditory or physical sensations associated with the implantation of a foreign body to complete device failure [3].

As reported in the literature, CI failures can be classified into three categories: hard, soft, and medical failures [4,5]. Medical failures are attributed to specific medical causes, such as the extrusion of the receiver–stimulator, migration of the electrodes, trauma, infection, or necrosis of the skin flap. In these cases, the devices are believed to be functioning normally but are removed for the described medical reasons [5,6].

There are two types of device failures: hard and soft failures. In hard failures, the CI is malfunctioning and fails integrity testing while still in vivo [4,7]. In soft failures, the inner device remains functional, but the patient experiences pain and changes in the quality of sounds [2]. According to the definition of the Soft Failures Consensus Statement in 2005, soft failure is a working diagnosis based on distinctive symptoms such as feelings of shock, clicking sounds, intermittence, or an unexplained progressive decrease in performance [8].

Since soft failures are defined when symptoms, clinical and audiological assessments, imaging, or device evaluation cannot identify the source of the failure, diagnosis and treatment become challenging and often delayed [4,6].

These CI-related adverse events impose a significant economic and emotional burden on CI recipients, healthcare providers, and manufacturers. Given their electronic nature, it is inevitable that CIs will wear out or fail over time, with a life expectancy estimated at over 20 years. Consequently [5], children receiving CIs are likely to require multiple re-implantations during their lifetime [7]. Therefore, it is crucial to enhance our understanding of the causes of CI adverse events to prevent or, at the very least, minimize the economic and emotional costs associated with these events.

The study aims to evaluate the clinical and audiological outcomes in long-term users of CIs, with a focus on hearing performance and device failures. These patients have been followed up for at least 10 years.

## 2. Materials and Methods

A retrospective review of the CI database was conducted for all patients with bilateral sensorineural hearing loss who received CIs between May 1997 and December 2012 at the University Hospital of Verona’s Otolaryngology Department.

Inclusion criteria

1. Bilateral hearing loss;
2. Uni- or bilateral CI implanted between 1998 and 2012 at our reference center;
3. Post-operative follow-up of a minimum of 10 years;
4. Standard surgical approach through a mastoidectomy and facial recess (posterior tympanotomy).

Exclusion criteria

1. Middle ear disease (for example, cholesteatoma, chronic otitis media);
2. Inner ear disease (for example, acoustic neuroma);
3. Single-side deafness (SSD);
4. Previous ear surgery (for example, stapedoplasty for otosclerosis).

The study population was divided into two groups: adults and children. Patients younger than 18 years were included in the children group.

Patients’ demographic and clinical data, age of implantation, CI characteristics, audiological skills, and complications were reviewed. The hearing thresholds at CI activation, one month post-surgery, and at the last available follow-up were also reviewed.

CI selection, surgery, and follow-up were performed after a standardized multidisciplinary assessment. The surgical procedure was performed under general anesthesia by an experienced operator, using a standard surgical approach through a mastoidectomy and facial recess (posterior tympanostomy). Electrodes were routinely inserted through round window insertion or cochleostomy in cases of ossified cochleas.

Devices from 4 manufacturers were used: Advanced Bionics, Cochlear, LAURA-Flex and MED-EL. CIs were activated one month after surgery. Implanted patients were reviewed at 3, 6, and 12 months during the first year and subsequently on an annual basis as per follow-up results or upon the patient’s or implant fitter’s request, as needed. Complications in this series were classified as either early (within the first week post-surgery) or late (more than 7 days post-surgery). Only major complications were considered. These include complications resulting in a serious medical condition (e.g., meningitis), requiring major surgical revision (e.g., electrode migration, facial nerve lesions, chronic infection, cholesteatoma, encephalocele), or causing permanent disability (e.g., device failure).

In 2017, the Association for the Advancement of Medical Instrumentation (AAMI) developed a standardized scheme for CI reliability reporting to ensure consistency in defining and categorizing device failures. In our study, all device failures were classified according to a modified version of the AAMI standard, incorporating both clinical assessments and manufacturer reports. This approach allowed us to systematically differentiate between hard failures, soft failures, and medical problems based on comprehensive clinical reasoning and diagnostic evaluations [9].

In hard failures, the CI malfunctions and fails integrity testing. In soft failures, the inner device remains functional, but the patient experiences changes in sound quality or decreased hearing. When CI failure was suspected, the work-up included a comprehensive assessment involving patient history, physical examination, and imaging (computed tomography, CT) to rule out array migration. Additionally, the evaluation encompassed re-programming of the device, replacement of external hardware, and integrity testing performed by the manufacturer. A team of ENT surgeons and audiologists conducted the clinical evaluation, with technical support obtained from the respective companies for assessing the integrity test.

Concerning audiological outcomes, we collected pure tone audiometry (PTA) values at activation and the last follow-up in patients without reimplantation. Additionally, values were gathered before and after CI revision surgery in patients experiencing device failure. Retrieving values for perception tests and language development was not possible, as they were neither available nor utilized in the majority of patients, particularly those with very extended follow-ups.

### Statistical Analysis

Mean and standard deviation were calculated for continuous parametric data. Data were analyzed using Stata Software (Version 18, © 1996–2023 StataCorp LLC, College Station, TX, USA). The chi-square test was used. A *p* < 0.05 was considered statistically significant.

Time to failure was calculated as years from the first surgery to reimplantation surgery. Kaplan–Meier plots and the log-rank test were used to illustrate survival curves for each implant brand.

The difference between pre- and post-reimplantation PTA thresholds was analyzed using a paired Student’s *t*-test. The correlations between PTA thresholds at implant activation (1 month post-surgery) and at the last follow-up were analyzed using Pearson’s product moment correlations.

For the analysis of survival curves and mean follow-up duration, the first revision surgery was considered a primary event, and the endpoint of observation was the last follow-up.

## 3. Results

Between May 1997 and December 2012, 193 patients, comprising 90 adults and 103 children with sensorineural hearing loss, received CIs at our institution. Their clinical and demographic characteristics are presented in Table 1.

The adult population consisted of 50 men and 40 women with a mean age of 45.8 ± 16.4 years (range: 19 to 75 years) at the time of implantation. The pediatric population comprised 55 boys and 48 girls with a mean age of 4.5 ± 4.5 years (range: 7 months to 17 years) at the time of implantation.

This series included 173 unilateral CIs (88 in adults and 85 in children) and 20 bilateral CIs (two in adults and 18 in children), including 15 cases of simultaneous implantation. The implant brands used were MED-EL (Innsbruck, Austria) (34.8%), Advanced Bionics (Southern California, USA) (32.6%), Cochlear (Sydney, Australia) (30.4%), and LAURA-Flex (Antwerp, Belgium) (2.2%) in adults, and Cochlear (68.6%), MED-EL (19.8%), and Advanced Bionics (11.6%) in children.

Since only two patients used LAURA-Flex devices, and both of them required revision, comparing them with devices from other manufacturers was impractical. Consequently, they were excluded from our statistical analysis. Therefore, data from 211 CIs (191 patients) were considered concerning failure and survival rates.

The mean postoperative follow-up period between the first implantation and the last control visit was 16.3 years (±4.53, range: 6–28 years). Specifically, the follow-up duration was 15.7 years (±4.4, range: 10–28 years) for adults and 16.9 years (±4.2, range: 10–25 years) for children. Fourteen (6.6%) major late postoperative complications were reported in this series, with no major early postoperative complications observed.

The global major complication rate was 6.6% (14 CIs). The device hard failure rate, calculated based on the total number of CIs, was 5.2% (11 patients), the principal cause of reimplantation, in agreement with the literature. Only two patients (0.9%) underwent surgery due to a medical problem (i.e., migration and extrusion of the electrode linked to head trauma) and another one (0.5%) due to a soft failure.

Table 2 displays demographic data for revision surgery patients. Out of the total, five patients (35.71%) were children and nine (64.29%) were adults. The time between the initial implantation and the revision surgery ranged from 4 to 21 years for the children’s group (mean 11.2 ± 6.6 years) and from 3 to 21 years for the adult group (mean 10.4 ± 6.8 years).

Thirteen patients were re-implanted simultaneously during the explantation in the same ear. Additionally, one patient underwent reimplantation in the contralateral (left) ear during the explantation, given a chronic infection on the right side.

All patients underwent the first CI surgeries by the same surgeon who performed the CI revision surgery.

The total reimplantation rate was then calculated for each manufacturer (Table 3), resulting in rates of 1.8%, 10.7%, and 13.6% for Cochlear, MED-EL, and Advanced Bionics, respectively.

There was no significant difference between device failure rates in the adult (10%) and children (4.1%) populations (*p* = 0.098).

**Figure 1:**
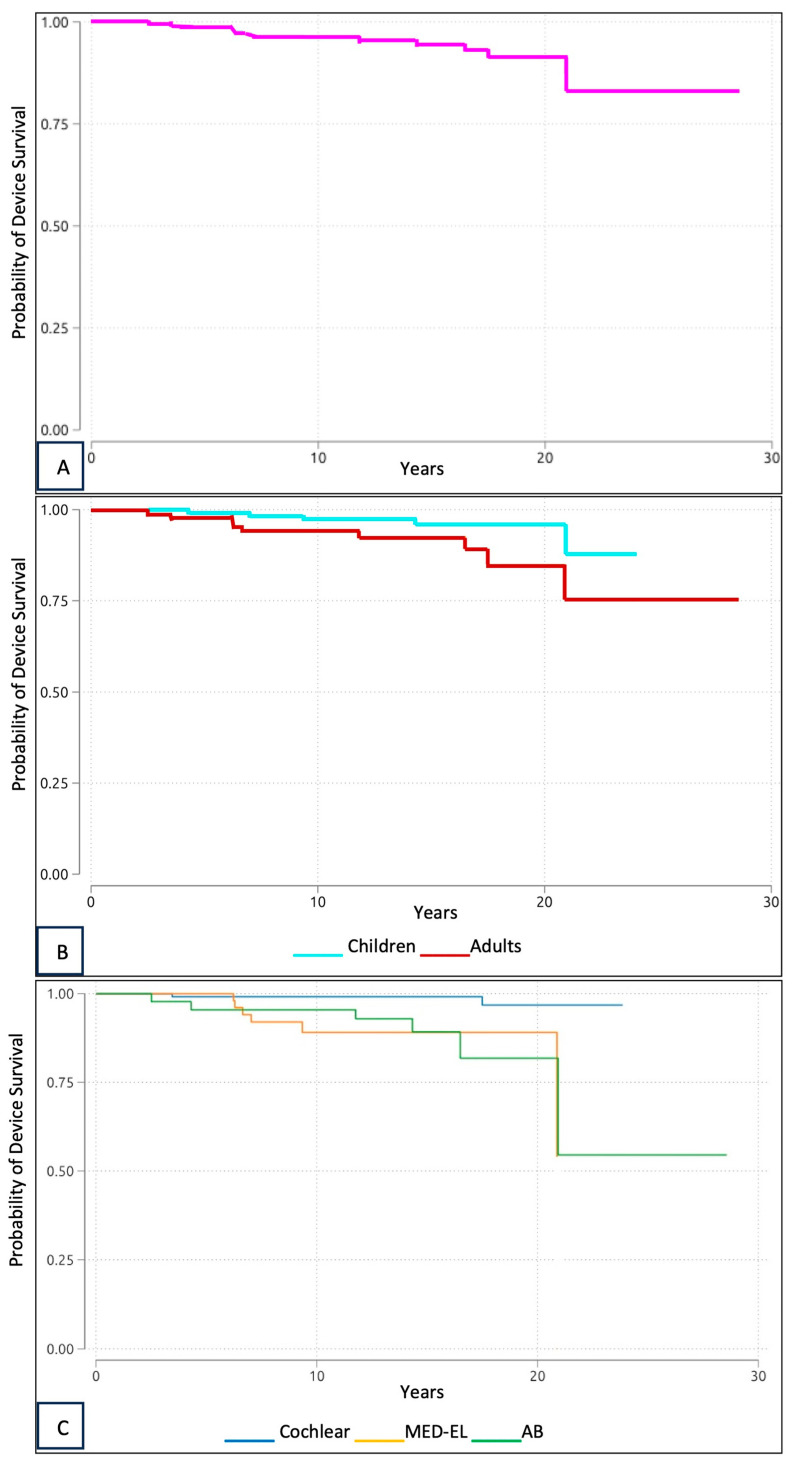
Kaplan–Meier survival analysis of CI devices. (**A**) Kaplan–Meier survival analysis of overall CI device survival. (**B**) Kaplan–Meier survival analysis of CI device survival in adults and children. (**C**) Kaplan–Meier survival analysis of CI device survival by manufacturer.

We conducted a survival analysis using the reviewed data, tracking the time from original implantation to reimplantation. The overall device survival rates were over 96% at 10 years and 91% at 20 years (Figure 1A). Device survival rate was also analyzed for the adult and pediatric populations (Figure 1B). In addition, the survival rates of the devices were analyzed separately for each brand (Figure 1C). There was a significant difference in failure rates among manufacturers (*p* = 0.000).

Finally, we reviewed the audiological results of all patients. In relation to the auditory thresholds of patients who did not undergo surgical revision (177 patients—197 CIs), thresholds were measured at major frequencies (0.5–1–2–4 kHz) for each patient at implant activation (1 month post-surgery) and at the last follow-up (with a mean duration of 15.84 years). The mean pure tone average (PTA) at implant activation was 35.5 ± 12.05 dB, and at the last follow-up, it was 31.07 ± 8.77 dB. From a statistical standpoint, a highly significant correlation was observed (*p* < 0.0001) between thresholds at implant activation and those at the last follow-up (Pearson correlation coefficient = 0.669).

In the group of patients who underwent CI revision surgery (14 patients—14 CIs), hearing thresholds with CIs for two patients (2/14 patients) before and after the revision surgery could not be retrospectively detected. However, the hearing threshold with a CI for the remaining 12 patients was present before and after the revision surgery. The PTA (0.5–4 kHz) was 41.93 ± 13.13 dB before the revision surgery and 34.79 ± 10.44 dB after the revision surgery.

We compared pre- and post-reimplantation PTA thresholds using a paired Student’s *t*-test and found no statistically significant difference (*p* = 0.154).

A total of 14 CI devices required reimplantation across three manufacturers. Among the Advanced Bionics implants, all six failed devices belonged to the HiRes90K model. Similarly, the two Cochlear implants that required reimplantation were both CI124 models. For MED-EL, six devices failed, with one case involving the PULSAR model and five cases related to the COMBI40+ model.

## 4. Discussion

The nature and rate of CI revision surgery have become increasingly significant due to the exponential growth in the number of patients receiving CIs, alongside an expansion in age range and indications. The cost of implantation, encompassing hardware, surgery, and rehabilitation, imposes a substantial burden on patients and clinics when revision surgery is necessary [10]. Being electronic devices, it is inevitable that CIs will wear out or fail over time. As reported by Mahtani et al., the life expectancy of a CI exceeds 20 years [7].

As can be seen from our study, major device failure was the most common indication for reimplantation (11 patients, 5.2%). Two patients (0.9%) were operated on for medical problems and one patient (0.5%) for soft failure. This finding aligns with what is reported in the literature [6,7].

Since the first extensive scientific report evaluating complications after CI surgeries by Cohen et al. in 1991, the overall complication rate has steadily decreased, thanks to improved surgical techniques and biocompatible implants [11]. The rate of surgical complications needing explantation and reimplantation reported in several studies varies from 1.8 to 12.3% [12] and from 1.2% to 15.1%, with a mean value of 7.6% [6].

The total rate of CI revision surgery in the present study was found to be 6.6%. Two recent review studies calculated overall revision rates of 6% and 4.6%, respectively [6,13]. In 2020, Kim et al. published a study that included patients undergoing CI surgery from October 2001 to March 2019. In this study, of 925 CI patients, 43 underwent revision surgery (4.6%). The mean total follow-up duration was 7.1 years. Lane et al. reviewed 804 CIs over 30 years, reporting a reimplantation rate of 2.9% and a device failure rate of 1.7% [6]. Their inclusion of recent implants with shorter follow-ups captured early failures in newer device models. In contrast, our study, with an average follow-up of 16.3 years (15.7 years for adults and 16.9 years for children), focuses on long-term device performance, offering insights into implant aging rarely explored in the existing literature. Similarly, Wang et al. analyzed 2827 CIs, reporting higher revision (8.3%) and failure rates (4.8%) [10]. While their broader dataset highlights early outcomes, our emphasis on extended follow-up provides critical data on long-term reliability and the potential for late device revisions. The EPIIC Registry tracked 5051 patients over a 5-year period, reporting a 1.9% explantation and reimplantation rate. While their multicenter design and large sample size offer robust epidemiological data, the shorter follow-up period limits insights into long-term device aging. Furthermore, due to the registry’s design, detailed information on specific implant models and brands was not available, making direct comparisons with device-specific longevity challenging [14].

By focusing on extended follow-up, our study provides essential data on device longevity, offering valuable guidance for clinicians in managing long-term outcomes—an aspect not fully addressed in previous studies.

In our study, a survival analysis was also performed to help determine the lifespan of the implanted devices. In the survival analysis, the overall device survival rates were more than 96% in 10 years and 91% in 20 years. These data imply that CIs remain safe and secure for many years. Furthermore, it is also evident from our curves that most failures occur after about 20 years, proving what is reported in the literature, i.e., that the life expectancy of CIs is longer than 20 years [7].

When the device failure rate was examined by brand, the rates for Cochlear, MED-EL, and Advanced Bionics were found to be 1.8%, 10.7%, and 13.6%, respectively. The decision to reimplant the devices was made on a case-by-case basis by a multidisciplinary CI team.

As mentioned earlier, LAURA-Flex devices were excluded from the statistical analysis. LAURA-Flex models exhibited the highest device failure rate, primarily due to being the oldest CIs used in this series, with one implanted in May 1997 and the other in March 1998. Additionally, this type of implant is now considered outdated and is no longer in use. Both patients with explanted LAURA-Flex devices were subsequently re-implanted with a CI from another brand, specifically MED-EL.

In this study, the survival rate was quite similar for MED-EL and Advanced Bionics. Notably, in our series, Cochlear exhibited the lowest device failure rate (1.8%) and revision rates, as well as the highest survival rates. However, it is important to interpret these findings with caution, as Cochlear was the most extensively utilized brand at our reference center, at least until 2012. The predominance of a single manufacturer in our sample, along with the relatively small number of reimplanted devices, may limit direct comparisons between manufacturers. This contrasts with other studies where MED-EL was more frequently employed, leading to different results with lower device failure and revision rates for MED-EL than for Cochlear [6,13,15,16].

Advanced Bionics implants exhibited a total reimplantation rate of 13.6%. This finding is consistent with Lane’s report, which indicates an overall device failure rate of 8.2% [6]. In our series, all the failures of Advanced Bionics were associated with HiRes90K devices, which were recalled in 2004 due to issues related to hermeticity and residual moisture in the stimulator’s internal component [6]. However, the relatively small number of Advanced Bionics implants in the present study contributes to high variability in device survival, making it challenging to draw definitive conclusions about these implants.

The overall revision rate was higher in the adult population (10%) compared to the pediatric group (4.1%) based on the total number of CIs (211). However, the difference in device failure rates between adults (10%) and children (4.1%) was not statistically significant (*p* = 0.098). This contrasts with recent studies, which have reported higher revision rates in children, likely due to skull growth and an increased risk of trauma [7,17,18,19]. Similarly, Van de Heyning et al. observed higher device failure rates in children, attributing this trend to increased physical activity and anatomical factors [17].

In our series, the adult population was defined as older than age 18 years at the time of implantation. Other studies, however, consider patients under 16 or 14 years of age as pediatric [6,10].

Farinetti et al. reported a global complication rate in the adult population significantly higher than that observed in the pediatric population (26.8% versus 14.9%). This difference was primarily associated with minor complications: children predominantly experienced infectious complications, while most cochleovestibular complications (such as tinnitus and vertigo) were observed in adults. Conversely, in terms of the major complication rate, no significant difference was observed between adults and children [1].

The management of CI complications and the need for revision surgery are on the rise due to the increasing number of implants. Helbig et al. demonstrated that it is possible to perform reimplantation while preserving residual hearing [20]. Furthermore, as reported by Manrique, cases requiring explantation and reimplantation of the CIs are considered safe procedures, with depth of insertion and speech perception results equaling or surpassing those of the initial implantation in most cases [21]. Several studies have indicated that speech perception performance often improves after reimplantation, and cochlear reimplantation itself does not have a negative effect on auditory outcomes [2,7,8,22,23]. Similarly, Yaar-Soffer et al. found that speech perception remained stable or improved in 84% of patients following revision CI, regardless of whether the reimplantation involved a device from the same or a different manufacturer [24]. Their findings further support the notion that cochlear reimplantation is a safe and effective procedure that does not negatively impact auditory outcomes, even in cases where manufacturer conversion occurs.

Another noteworthy finding that emerges from our case series is the statistical significance (*p* < 0.0001) between PTA thresholds at implant activation and those at the last follow-up (Pearson correlation coefficient = 0.669). Therefore, CIs exhibit a performance decrement during long-term follow-up. As we have seen, the decrement in auditory performance falls within Balkany’s definition of “soft failure” from 2005 [8]. Many soft failures may initially be underestimated or misdiagnosed, and, as time progresses, CIs experience a significant loss of performance and ‘get old’.

Layfield et al. documented a substantial incidence of performance decrement/adverse reactions (PD/AR) in their case series, reaching 34.3% [25]. They assert that performance decrement is a dynamic category that might reflect patients with consistently suboptimal outcomes. With the growing diversity of patients receiving implants, we may observe a simultaneous increase in varying outcomes. Consequently, the wide range of reported performance decrement rates is likely due to the expansion of implantation criteria, differences in institutional protocols, and inconsistencies in how performance decrement is defined across studies.

In conclusion, while CI is generally considered a safe procedure, the occurrence of device and medical failures may necessitate revision surgeries. Like any surgical intervention, some patients may experience postoperative complications. Revision surgery, although deemed safe, can impose an emotional burden on patients and their families. Therefore, it is crucial for healthcare professionals, including doctors, speech therapists, CI patients, their parents, and other stakeholders, to be aware of the potential for revision surgeries [26,27,28,29,30]. Furthermore, early diagnosis and treatment of CI failure are crucial and particularly challenging, especially for children, for whom hearing plays a fundamental role in language development [18].

Our experience suggests that CIs demonstrate durability; however, as with any technological device, failures can occur even after many years. Notably, our data provide more reliable insights into long-term follow-ups on patients implanted for at least 10 years.

To date, other studies have examined the failure rate of CIs in long-term follow-up. However, to our knowledge, this is the first study to focus only on implants with a minimum follow-up of 10 years. Therefore, we have obtained more specific data regarding potential long-term complications of CIs.

### Limitations of the Study

This study has several potential sources of bias. Its monocentric and retrospective nature may have led to the omission of contributing factors, particularly complications that might have been underreported. Due to the age of some clinical records, complete data on all implant models were not available; however, this did not impact the analysis of long-term device performance and reliability. Additionally, certain surgical factors that could have influenced outcomes, such as difficulties in electrode re-insertion, were not consistently recorded.

Furthermore, the relatively small sample size, particularly when comparing manufacturers, limits the generalizability of the findings.

The 10-year minimum follow-up allowed us to assess CI aging and long-term reliability, providing valuable insights into device lifespan and the need for revisions over time. However, this focus may have resulted in the exclusion of early device failures, which typically occur within the first 5 years post-implantation, as well as newer CI models. Despite this, our study offers unique long-term data that are rarely addressed in the existing literature, contributing valuable information for both patients and clinicians.

Finally, since some patients came from other Italian regions, late complications may have been managed at outside hospitals and, therefore, not reflected in our dataset.

## 5. Conclusions

Unilateral or bilateral CI surgery is a life-changing surgery that improves patients’ social lives and provides academic and professional gain. It is a safe surgical technique for hearing rehabilitation associated with a low rate of severe complications. Moreover, it is a lifelong device. However, the technology is constantly evolving. It is therefore essential to provide thorough counseling to patients, or to parents in the case of pediatric patients, to ensure they fully understand the potential long-term outcomes of CIs, including the possibility of device aging and the need for future revision surgeries.

## Figures and Tables

**Table 1:** Demographic and clinical characteristics of the 193 patients who received CIs between 1997 and 2012. Patients under the age of 18 are categorized as children.

	Adults		Children	
	Total	%	Total	%
**Gender**				
Male	50	55.6	55	53.4
Female	40	44.4	48	46.6
**Implanted side**				
Right	57	63.4	89	86.5
Left	33	36.6	14	13.5
**Mode of implant**				
Unilateral	88	97.8	85	82.5
Bilateral	2	2.2	18	17.5
**Manufacturers**				
MED-EL	32	34.8	24	19.8
Cochlear	28	30.4	83	68.6
Advanced Bionics	30	32.6	14	11.6
LAURA-Flex	2	2.2	0	0

**Table 2:** Demographic data for revision surgery patients. F: female; M: male; L: left; R: right; AB: Advanced Bionics.

Patients	Sex	Side of Implant	Duration (Years)	Brand	Type of Failure
**Adults**					
**A1**	F	R	12	AB	Hard failure
**A2**	M	L	6	MED-EL	Hard failure
**A3**	F	L	6	MED-EL	Hard failure
**A4**	F	R	17	AB	Medical problem
**A5**	M	L	3	AB	Hard failure
**A6**	F	L	3	Cochlear	Hard failure
**A7**	F	R	6	MED-EL	Hard failure
**A8**	M	L	21	MED-EL	Hard failure
**A9**	M	R	18	Cochlear	Hard failure
**Children**					
**C1**	F	R	7	MED-EL	Hard failure
**C2**	F	L	21	AB	Soft failure
**C3**	M	R	4	AB	Hard failure
**C4**	M	L	14	AB	Medical problem
**C5**	F	R	10	MED-EL	Hard failure

**Table 3:** Total reimplantation rate calculated for each manufacturer. AB: Advanced Bionics.

	MED-EL	Cochlear	AB
**Adults**			
Number of CIs	32	28	30
Number of reimplantations	4	2	3
Reimplantation rate (%)	12.5	7.1	10
**Children**			
Number of CIs	24	83	14
Number of reimplantations	2	0	3
Reimplantation rate (%)	8.3	0	21.4
**Total**			
Number of CIs	56	111	44
Number of reimplantations	6	2	6
Reimplantation rate (%)	10.7	1.8	13.6

## Data Availability

Data is contained within the article.

## References

[1] Farinetti A., Ben Gharbia D., Mancini J., Roman S., Nicollas R., Triglia J.M. (2014). Cochlear implant complications in 403 patients: Comparative study of adults and children and review of the literature. Eur. Ann. Otorhinolaryngol. Head Neck Dis..

[2] Gumus B., İncesulu A.S., Kaya E., Kezban Gurbuz M., Ozgur Pınarbaslı M. (2021). Analysis of cochlear implant revision surgeries. Eur. Arch. Otorhinolaryngol..

[3] Tambyraja R.R., Gutman M.A., Megerian C.A. (2005). Cochlear implant complications: Utility of federal database in systematic analysis. Arch. Otolaryngol. Head Neck Surg..

[4] Yosefof E., Hilly O., Ulanovski D., Raveh E., Attias J., Sokolov M. (2021). Cochlear implant failure: Diagnosis and treatment of soft failures. Acta Otorhinolaryngol. Ital..

[5] Causon A., Verschuur C., Newman T.A. (2013). Trends in cochlear implant complications: Implications for improving long-term outcomes. Otol. Neurotol..

[6] Lane C., Zimmerman K., Agrawal S., Parnes L. (2020). Cochlear implant failures and reimplantation: A 30-year analysis and literature review. Laryngoscope.

[7] Mahtani S., Glynn F., Mawman D.J., O’Driscoll M.P., Green K., Bruce I., Freeman S.R., Lloyd S.K. (2014). Outcomes of cochlear reimplantation in adults. Otol. Neurotol..

[8] Balkany T.J., Hodges A.V., Buchman C.A., Luxford W.M., Pillsbury C.H., Roland P.S., Shallop J.K., Backous D.D., Franz D., Graham J.M. (2005). Cochlear implant soft failures consensus development conference statement. Otol. Neurotol..

[9] Association for the Advancement of Medical Instrumentation Cochlear Implant Committee (2017). Cochlear Implant Systems: Requirements for Safety, Functional Verification, Labeling, and Reliability Reporting. American National Standards Institute.

[10] Wang J.T., Wang A.Y., Psarros C., Da Cruz M. (2014). Rates of revision and device failure in cochlear implant surgery: A 30-year experience. Laryngoscope.

[11] Cohen N.L., Hoffman R.A. (1991). Complications of cochlear implant surgery in adults and children. Ann. Otol. Rhinol. Laryngol..

[12] Petersen H., Walshe P., Glynn F., McMahon R., Fitzgerald C., Thapa J., Simoes-Franklin C., Viani L. (2018). Occurrence of major complications after cochlear implant surgery in Ireland. Cochlear Implants Int..

[13] Kim S.Y., Kim M.B., Chung W.H., Cho Y.S., Hong S.H., Moon I.J. (2020). Evaluating Reasons for Revision Surgery and Device Failure Rates in Patients Who Underwent Cochlear Implantation Surgery. JAMA Otolaryngol. Head Neck Surg..

[14] Hermann R., Coudert A., Aubry K., Bordure P., Bozorg-Grayeli A., Deguine O., Eyermann C., Franco-Vidal V., Godey B., Guevara N. (2020). The French National Cochlear Implant Registry (EPIIC): Cochlear explantation and reimplantation. Eur. Ann. Otorhinolaryngol. Head Neck Dis..

[15] Soloperto D., Dallari V., Molteni G. (2023). How I do it: Cochlear Osia 2 System surgery placement. Eur. Arch. Otorhinolaryngol..

[16] Di Maro F., Carner M., Sacchetto A., Soloperto D., Marchioni D. (2022). Frequency reallocation based on cochlear place frequencies in cochlear implants: A pilot study. Eur. Arch. Otorhinolaryngol..

[17] Van de Heyning P., Atlas M., Baumgartner W.D., Caversaccio M., Gavilan J., Godey B., Gstöttner W., Hagen R., Yongxin L., Karltorp E. (2020). The reliability of hearing implants: Report on the type and incidence of cochlear implant failures. Cochlear Implants Int..

[18] Cullen R.D., Fayad J.N., Luxford W.M., Buchman C.A. (2008). Revision cochlear implant surgery in children. Otol. Neurotol..

[19] Sorrentino T., Coté M., Eter E., Laborde M.L., Cochard N., Deguine O., Fraysse B. (2009). Cochlear reimplantations: Technical and surgical failures. Acta Otolaryngol..

[20] Helbig S., Rajan G.P., Stöver T., Lockley M., Kuthubutheen J., Green K.M. (2013). Hearing preservation after cochlear reimplantation. Otol. Neurotol..

[21] Manrique-Huarte R., Huarte A., Manrique M.J. (2016). Surgical findings and auditory performance after cochlear implant revision surgery. Eur. Arch. Otorhinolaryngol..

[22] Migirov L., Taitelbaum-Swead R., Hildesheimer M., Kronenberg J. (2007). Revision surgeries in cochlear implant patients: A review of 45 cases. Eur. Arch. Otorhinolaryngol..

[23] Carner M., Sacchetto A., Bianconi L., Soloperto D., Sacchetto L., Presutti L., Marchioni D. (2019). Endoscopic-Assisted Cochlear Implantation in Children with Malformed Ears. Otolaryngol. Head Neck Surg..

[24] Yaar-Soffer Y., Shapira Y., Sagiv D., Yakir Z., Wolfovitz A., Henkin Y. (2024). Revision Cochlear Implantation With Device Manufacturer Conversion: Surgical Outcomes and Speech Perception Performance. Otolaryngol. Head Neck Surg..

[25] Layfield E., Hwa T.P., Naples J., Maina I., Brant J.A., Eliades S.J., Bigelow D.C., Ruckenstein M.J. (2021). Failure and Revision Surgery After Cochlear Implantation in the Adult Population: A 10-year Single-institution Retrospective and Systematic Review of the Literature. Otol. Neurotol..

[26] Soloperto D., De Cecco F., Confuorto G., Dallari V., Nocini R., Carner M., Sacchetto L. (2024). Hearing rehabilitation in children with malformed ears: The endoscopic-assisted approach for cochlear implantation. Am. J. Otolaryngol..

[27] Chen J., Chen B., Shi Y., Li Y. (2022). A retrospective review of cochlear implant revision surgery: A 24-year experience in China. Eur. Arch. Otorhinolaryngol..

[28] Dallari V., Apa E., Monzani D., Genovese E., Marchioni D., Soloperto D., Sacchetto L. (2022). Cochlear Implantation Following Transcanal Infrapromontorial Approach for Vestibular Schwannoma: A Case Series. Audiol. Res..

[29] Ikeya J., Kawano A., Nishiyama N., Kawaguchi S., Hagiwara A., Suzuki M. (2013). Long-term complications after cochlear implantation. Auris Nasus Larynx.

[30] Weichbold V., Zelger P., Galvan O., Muigg F. (2023). 5-Year Observation Period of Quality of Life After Cochlear Implantation. Otol. Neurotol..

